# Pseudorabies Virus Infections

**DOI:** 10.3390/pathogens10060719

**Published:** 2021-06-08

**Authors:** Barbara G. Klupp

**Affiliations:** Institute of Molecular Virology and Cell Biology, Friedrich-Loeffler-Institut, 17493 Greifswald-Insel Riems, Germany; barbara.klupp@fli.de

Suid alphaherpesvirus 1 (SuHV-1), better known as Pseudorabies virus (PrV), an alphaherpesvirus of swine, is the causative agent of Aujeszky’s Disease. In the previous century, epidemics of Aujeszky´s disease have led to enormous economic losses in pig farms and an efficient vaccine was urgently needed. The highly attenuated “K61” strain, which was isolated after numerous serial passages on different cell cultures, published by A. Bartha in 1961, proved to be the crucial milestone in the fight against this disastrous disease. The Bartha K61 strain is still one of the best live virus vaccines in use and “celebrates” its 60th anniversary this year (2021). In the concise review by J.L. Delva et al. [1] the history and the molecular characteristics as well as the immunogenic differences to wildtype strains is summarized and the efficacy of PrV Bartha K61 as neuronal tracer as well as vector vaccine is discussed. In combination with suitable serological assays, Bartha K61 was developed into the first marker vaccine differentiating infected from vaccinated animals (DIVA), thereby serving as the blueprint in modern animal vaccinology. In the entertaining review by T.C. Mettenleiter [2], the history of the discovery of PrV as the causative agent of the disease is described, honoring the great achievements of A. Aujeszky and offering vivid insights into former research. Although the natural host of PrV is swine, it can infect a wide variety of different mammals, usually with a fatal outcome. In a comprehensive comparative review article, J. Sehl and J.P. Teifke [3] summarize the clinical–pathological findings of PrV infection in different animal species after natural or experimental infection highlighting the similarities but also differences in the route of infection, clinical presentation, antigen distribution as well as lesion patterns. Although pruritus is the classical sign of PrV infection in non-host species, which led to the term “mad itch disease”, it usually does not occur in infected pigs. However, the molecular basis for this neurological itch is still not well understood. The review of K. Laval and L.W. Enquist [4] provides detailed insights, discussing the recent findings on the neuro-inflammatory responses and their impact on other alphaherpesvirus induced neuropathies, for example those following infection with varicella zoster virus. A better understanding will help to develop new therapeutics and guide research on other neuropathies, such as multiple sclerosis [4].

Although PrV is eradicated from domestic pigs in many Western countries, the virus is still circulating in wild boar populations coupled with infrequent infections of hunting dogs and the risk of reintroduction into farmed animals. Surveillance is thus still an important issue and discussed in three original articles [5,6,7]. Drugs to fight PrV infections are not available but natural compounds, as Germacrone, are tested for their effect as putative therapeutics [8]. 

PrV is not only prominent as animal pathogen but, together with herpes simplex virus 1, represents the most intensively studied and best understood alphaherpesvirus at the molecular level. Studies on PrV repeatedly pioneered research on basic principles of herpesvirus replication. Data on the complex transcriptome [9], the functional role of N-linked glycans of glycoprotein gB [10] and an unbiased approach to uncover the still unknown signaling network of the pUS3 protein kinase [11] will stimulate further research on the molecular specifics of PrV and other (alpha-)herpesviruses.
